# Economic evaluation of brief cognitive behavioural therapy for social activation in recent-onset psychosis

**DOI:** 10.1371/journal.pone.0206236

**Published:** 2018-11-12

**Authors:** Ben F. M. Wijnen, Karin Pos, Eva Velthorst, Frederike Schirmbeck, Hoi Yau Chan, Lieuwe de Haan, Mark van der Gaag, Silvia M. A. A. Evers, Filip Smit

**Affiliations:** 1 Centre for Economic Evaluation, Trimbos Institute, Netherlands Institute of Mental Health and Addiction, Utrecht, the Netherlands; 2 CAPHRI School for Public Health and Primary Care, Department of Health Services Research, Maastricht University, Maastricht, the Netherlands; 3 Department of Psychiatry, Early Psychosis Section, Academic Medical Centre, University of Amsterdam, Amsterdam, The Netherlands; 4 Department of Psychiatry, Icahn School of Medicine at Mount Sinai, New York, United States of America; 5 Seaver Autism Center for Research and Treatment, Icahn School of Medicine at Mount Sinai, New York, United States of America; 6 Department of Clinical Psychology, VU University, Amsterdam, The Netherlands; 7 Parnassia Psychiatric Institute, The Hague, The Netherlands; 8 Department of Epidemiology and Biostatistics, Amsterdam Public Health research institute, VU University medical center, Amsterdam, the Netherlands; TNO, NETHERLANDS

## Abstract

**Background:**

In schizophrenia spectrum disorders, negative symptoms (e.g. social withdrawal) may persist after initial treatment with antipsychotics, much affecting the quality of life (QOL) of patients. This health-economic study evaluated if a dedicated form of cognitive behaviour therapy for social activation (CBTsa) would reduce negative symptoms and improve QOL in an economically sustainable way.

**Methods:**

A health-economic evaluation was conducted alongside a single-blind randomised controlled trial in two parallel groups: guideline congruent treatment as usual (TAU; n = 50) versus TAU augmented with adjunct CBTsa (n = 49). Outcomes were PANSS negative symptom severity and EQ-5D quality adjusted life years (QALYs) gained. The health-economic evaluation was conducted both from the societal and the health sector perspective.

**Results:**

Both conditions showed improvement in the respective outcomes over the follow-up period of six months, but QALY gains were significantly higher in the CBTsa condition compared to the TAU condition. Treatment response rate (i.e. ≥ 5-point decrease on the PANSS) was not significantly different. However, the add-on CBT intervention was associated with higher costs. This did not support the idea that CBTsa is a cost-effective adjunct. Various sensitivity analyses attested to the robustness of these findings.

**Conclusions:**

In the Dutch context where TAU for psychosis is guideline congruent and well implemented there appears no added value for adjunct CBTsa. In other settings where the treatment for the schizophrenia spectrum disorders solely relies on antipsychotics, add-on CBTsa may lead to clinically superior outcomes, but it should still be evaluated if adjunct CBTsa therapy is a cost-effective alternative.

**Trial registration:**

ClinicalTrials.gov registry under NCT03217955.

## 1. Introduction

People with schizophrenia have been reported to have a diminished capacity for learning, working, self-care, interpersonal relationships and general living skills [1, 2]. Schizophrenia spectrum disorders entail significant cost to patients in terms of personal suffering, but also on the caregiver as a result of the shift of burden of care from hospital to families in Europe [3]. Furthermore, these disorders significantly increases the healthcare costs, e.g. by hospitalizations, need for long-term psychosocial support, and life-long productivity losses [3]. A study of Wu et al. (2005) concluded that even the lowest prevalence estimate of schizophrenia represents an excess cost of considerable magnitude, equivalent to 39.9 billion U.S. dollars [1]. In the Netherlands about 2% of the total health care budget is spent on the treatment of schizophrenia [4]. Healthcare costs are only a minor part of the total expenditure. It has been estimated that the indirect costs stemming from productivity losses contribute to 50%–85% of the total costs associated with schizophrenia [5, 6]. Moreover, it has been demonstrated that negative symptoms in patients with schizophrenia are associated with higher total costs compared to patients without negative symptoms, especially with regard to healthcare costs (i.e., primary care) [7].

Cognitive deficits and negative symptoms of schizophrenia are highly associated to social dysfunctions. Remediation of cognitive deficits in early psychosis has only limited success with an effect-size of 0.13 [8]. Social skills training is the most effective treatment of negative symptoms [9] and CBT has some promising results with targeting dysfunctional expectancies [10, 11]. Moreover, it has been shown that women with schizophrenia and patients with a low level of conviction in their delusions are most likely to respond to (brief) CBT [12]. The intervention in this study combines social activation with a focus on dysfunctional expectancies of their own performance in these patients. As the persistence of cognitive deficits and negative symptoms is well-known, it may be interesting to see whether existing interventions with small effects can be cost-effective.

As extra care often comes with additional treatment costs it is important to determine how much benefits one receives from the added CBT intervention. To this extent, economic evaluations are designed to provide a quantitative insight in the added value of treatments and are becoming a common requirement for reimbursement decisions [13]. In an economic evaluation, one compares both the costs and effects of two (or more) alternative treatments or interventions in a systematic manner. Hence, it is possible to examine which alternative is most efficient [14].

The economic evaluation in this study was conducted to examine the added value of Cognitive Behavioural Therapy for social activation (CBTsa) in recent onset schizophrenia spectrum disorders as compared to treatment as usual (TAU) alone. The cost-effectiveness and cost-utility analyses will be conducted from both the health care and societal perspective with a last follow-up at 6 months post baseline. In addition, we examined whether there were any subgroups in which the intervention is particularly cost-effective using incremental net-benefit regression analyses.

## 2. Methods

**Fig 1:**
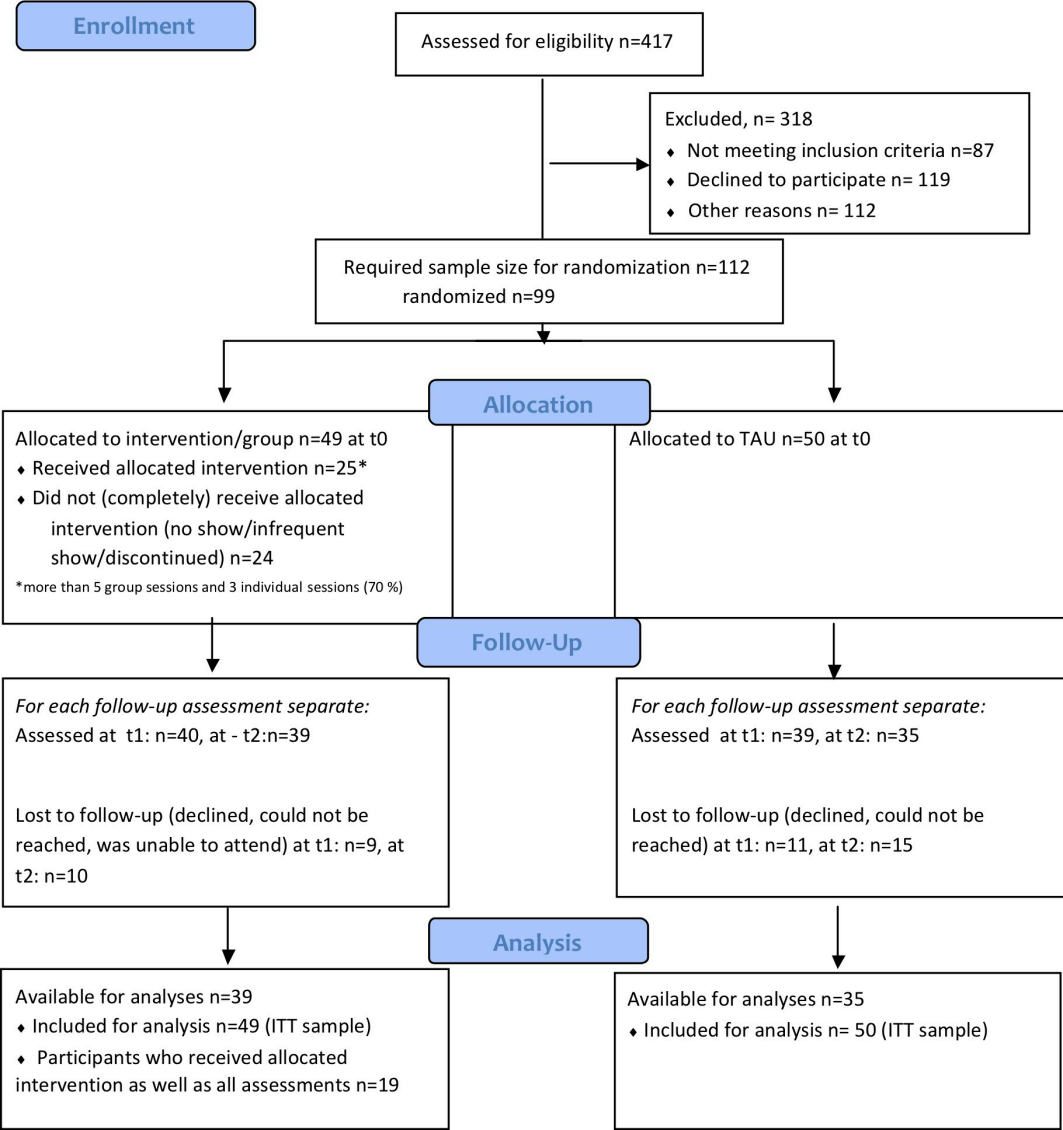
Overview of the design of the study and outcome assessments.

This economic evaluation was embedded in a single blind randomized controlled trial (RCT) directed at patients with a recent onset schizophrenia spectrum disorder. Patients were randomly allocated to the intervention group (CBTsa) or treatment as usual (TAU). Patients were stratified by sex, because women with recent onset schizophrenia have a better prognosis and may respond differently to CBTsa [15, 16]. Measurements were conducted at baseline (t0), post-treatment at 3 months (t1), and a follow-up at 6 months (t2) (i.e. 3 months after the end of treatment). A flowchart of the study can be found in Fig 1. The study was approved by the Medical Ethics Committee of the Academic Medical Center Amsterdam. The trial is registered at ClinicalTrials.gov registry under NCT03217955.

### 2.1 Study population

Participants recruited for the study were either hospitalized or attending day-treatment or receiving outpatient care at one of the following treatment centres: Academic Medical Center Amsterdam, Arkin Institute in Amsterdam, InGeest outpatient psychiatric service in Amsterdam, Altrecht ABC team in Utrecht, and Centrum First Psychosis Parnassia in The Hague. To be included in the study, patients had to be between 18 and 36 years old and diagnosed with DSM-IV-TR [17] schizophrenia or a related disorder with onset of their first psychotic episode < 4 years prior to inclusion. Patients with a comorbid diagnosis of a Bipolar Disorder or Autism Spectrum Disorder were not included in this study. Only participants with at least a mild level of social withdrawal behaviour, defined as a score of ≥ 3 on apathy/social withdrawal as measured with the negative scale of the PANSS [18], or ≥ 2 on the social isolation items of the Brief Negative Symptom Scale (BNSS) [19] were included. When negative symptoms were primarily the result of positive symptoms (e.g. withdrawal due to paranoid delusions) subjects were not included, as the therapy was especially tailored to target primary social withdrawal.

Based on a study of Grant et al. (2012), an effect size of (at least) .66 was anticipated [11]. With an alpha of 0.5; power = 80%; effect-size .66, this would imply that 36 participants per group are required to detect a true treatment difference. Taking into account an expected dropout rate of 20%, we would need to include 90 participants, i.e. 45 per group. To take into account the ‘variance inflation’ factor (due to our multi-center design), we calculated the Intra Class Correlation Coefficient of the PANSS negative symptom scores of a study targeting a similar population and setting. The ICC in this study was .146. Following the literature, we then used the following formula: 1 + (m- 1) x ICC, where m = number of participating centers. Hence, the required sample size for this study to achieve a power of 80% was estimated to be 112 (56 per condition).

### 2.2 Intervention

Patients allocated to the CBTsa-group received CBTsa in addition to TAU. The CBTsa therapy consisted of two components: 1) group sessions for 4 weeks, two sessions per week, 60 minutes per session, two trainers (a CBT therapist and a CBT assistant) in groups of eight participants per group; and 2) individual sessions (crystallizing learned skills, focus on individual needs) during 6–8 weeks, one session per week, 45 minutes per session.

The CBTsa therapy was based on accumulating evidence that dysfunctional beliefs in conjunction with neurocognitive impairments can impede functioning [10]. The core assumption of this therapy was that modifying dysfunctional beliefs may lead to increased engagement in constructive social activity in individuals with prominent negative symptoms [20].

The group sessions included psycho-education, peer support, buddy-forming, social goal setting, breaking goals down into steps and planning them, find obstacles and dysfunctional cognitions, behavioural experiments, and imagery for executing goal steps. The individual sessions focused on individual case formulations (including the person's main dysfunctional beliefs and associated behaviours), continuation of working on social goals and countering obstacles to these goals. In addition, psycho-education about symptoms, the role of cognition / beliefs and consequences of current behaviour were addressed.

Sessions were adapted to fit the young population (e.g. to focus on specific forms of impaired social functioning) and treatment session time and duration were adapted to the needs of individual patients.

### 2.3 Treatment as usual

Patients in the TAU condition received treatment as usual (without CBTsa) at one of the participating centres in which they were hospitalized, attended day-treatment or outpatient treatment. Across sites, TAU consisted of early intervention programs where patient’s symptoms, functioning and medication use were monitored for 3 years. At minimum, treatment as usual consisted of antipsychotic medication and supportive therapy. Additionally standard care for patients with psychotic disorders could involve psycho-education, family support, physical health care, psycho-motor therapy and/or vocational therapy [21]. The latter includes Individual Placement and Support (IPS), which supports patients to achieve employment and reintegration. The teams of the participating psychiatric services included psychiatrists, psychologists, psychiatric nurses and social workers. Participants in the TAU condition were not allowed to receive any form of CBT or any intervention that was specifically focused on social activation.

### 2.4 Outcomes

To determine the (clinical) effectiveness the Positive and Negative Syndrome Scale for Schizophrenia (PANSS) was used [22]. The PANSS is a 30-item instrument that provides balanced representation of positive and negative symptoms and gauges their relationship to one another and to global psychopathology. The PANSS consists of three sub-scales: a positive syndrome scale (7 items), a negative syndrome scale (7 items) and a general psychopathology scale (16 items). For the cost-effectiveness analysis (CEA), the central clinical end-term was treatment response, defined as a decrease on the PANSS negative symptom score (with a 5-point decrease meaning a clinical relevant change; arbitrary chosen). For the cost-utility analysis (CUA), the EuroQol 5 dimensions 5 levels (EQ-5D-5L) was used to assess health-related quality of life (QOL) [23]. The EQ-5D-5L is a 5-item questionnaire tapping into 5 dimensions: mobility, self-care, usual activities, pain/discomfort and anxiety/depression. Utilities were derived from the EQ-5D-5L using the Dutch tariffs [24]. A utility represents the value of a patient’s health state and is measured on a continuous scale anchored between 0 and 1, in which 0 means worst imaginable health state and 1 perfect health. Utilities are used to calculate quality adjusted life years (QALYs) by multiplying the years spend in a specific health state by the utility of that health state. QALYs were calculated using the area under the curve method [14].

### 2.5 Costs

This economic evaluation was performed according to the Dutch guidelines for economic evaluations [25] and the Consolidated Health Economic Evaluation Reporting Standards were used to report the outcomes of the health-economic evaluation [26].

Resource use owing to health care uptake was measured using the Trimbos/iMTA Questionnaire Costs associated with Psychiatric illness (TiC-P) [27].

Costs were divided into four categories: intervention costs, healthcare sector costs, costs for patient and family, and productivity costs. Intervention costs were calculated based on the number of group sessions and individual sessions and associated staff, overhead and patient time costs. Standardized cost prices from the Dutch manual for costing, and, if not available, mean cost prices from the providers were used as unit cost prices [25]. To determine the costs of medication, the website of the Dutch healthcare institute for the cost of pharmaceuticals (www.medicijnkosten.nl) was used. Productivity losses were estimated using the friction cost approach as recommended by the Dutch guidelines. In the Netherlands, a friction period of 85 days is recommended for economic evaluations [25]. The friction period is the time until another worker from the pool of unemployed has fully replaced the individual who is absent due to an illness [28]. By implication, the cost stemming from productivity losses through absenteeism seize to exist after the friction period. Patients’ time and informal care was valued using the proxy good method using the average hourly wage of domestic help as a proxy.

All costs were indexed for the year 2015. Discounting of costs nor and effects was not carried out, because the study follow-up was less than one year.

### 2.6 Analyses

All analyses were performed in accordance with the intention to treat principle. In agreement with the Consolidated Standards of Reporting Trials [29], possible baseline differences were not statistically tested; instead we used clinical and economic judgment to see if baseline differences (if any) were deemed relevant. To determine the clinical effect of the treatment over time (i.e. on responder rate and QALY) logistic regression (for responder rate) and linear regression (for QALYs) was used. A detailed elaboration on the clinical effects can be found in another paper by our group [30].

For the economic evaluation the following data-analytic approach was adopted. PANSS-scores, EQ-5D-5L scores, total health care costs, total patients & family costs and total productivity costs at each follow-up were imputed using multiple imputation (5 times). Imputation was based on age, gender, use of antipsychotic medication, baseline PANSS-scores, randomization group, health care, patients & family and productivity costs at each time point (for cost data only) and EQ-5D-5L scores at each time point (for EQ-5D data only). Multiple imputation was done using predictive mean matching in which “real” observed values from similar cases are imputed instead of imputing regression estimates to account for non-normality of the cost and EQ-5D-5L data [31].

The incremental cost-effectiveness ratio (ICER) was calculated as the extra costs per additional treatment responder (defined as a ≥ 5-point decrease on the PANSS negative symptom score). To determine the costs per QALY gained, the incremental cost-utility ratio (ICUR) was calculated using utility values derived from the EQ-5D-5L. ICURs were calculated by dividing the incremental costs by gaining one QALY. Seemingly unrelated regression equations (SURE) were bootstrapped (5 000 times) to allow for correlated residuals of the cost and utility equations and plotted on a cost-effectiveness plane (CE-plane). Bootstrapping is a non-parametric way to repeatedly conduct an analysis by resampling, with replacement, from the observed data [32]. For decision-making purposes, a cost-effectiveness acceptability curve (CEAC) was plotted. A CEAC plots the likelihood that the new intervention is cost-effective for various willingness-to-pay (WTP) ceilings for gaining a QALY. In the Netherlands, ceiling ratios can be roughly estimated to be €20 000–80 000 per QALY depending on the severity of the disease or disorder [33].

To determine subgroups in which the intervention was particularly cost-effective incremental net-benefit regression (INBRA) was used. INBRA is essentially a regression analysis were the treatment dummy, a prognostically relevant population characteristic (e.g. Dutch vs non-Dutch ethnicity) and their interaction are regressed on net-benefits. Net-benefits, NB, were defined as: NB = (E * λ)–C, where E are the effects per patient (QALYs), C are the costs per patient and λ is the willingness to pay for a unit of effect (i.e. €20 000 and €80 000 per QALY gained). INBRAs were conducted using sex, randomization group, ethnicity, educational level, age and social class as independent variables. Analyses were performed using STATA 14, and Microsoft Excel 2010.

### 2.7 Sensitivity analysis

To assess the robustness of our findings one-way sensitivity analyses were performed. In the main analysis results were not adjusted for baseline differences. However, we noticed some differences regarding the use of antipsychotic medication at baseline. Hence, a SURE model including baseline antipsychotic medication use was used to examine the effect of antipsychotic use. In addition, analyses were performed from a health care perspective, instead of the societal perspective as was done in the main analysis.

## 3. Results

### 3.1 Sample at baseline

Table 1 presents the sample at baseline. In total, 99 patients were included in the study. Of these, 49 were assigned to the intervention condition. Mean age at in the CBTsa condition was 25.14 years (SD = 4.47) and 25.72 years (SD = 4.44) in the TAU condition. In the CBTsa condition 75.5% was male and in the TAU condition 86% was male. At baseline, patient groups appeared to differ substantially regarding their use of antipsychotic medication, but both groups were comparable with respect to their DSM-IV symptom level, with paranoid symptoms, psychotic symptoms, and affective symptoms being the most common. The mean number of followed CBTsa-group sessions by patients was 6.4 (SD = 2.3); range = 0–8, and of individual sessions 3.9 (SD = 2.8); range = 0–8.

**Table 1:** Demographic and clinical characteristics of the experimental and control groups at baseline.

		CBTsa-group (N = 49)	TAU group (N = 50)
Demographics		Mean (SD)	Mean (SD)
Age		25.14 (4.47)	25.72 (4.44)
Sex ratio male/female		37/12	43/7
Ethnicity % minority		69.4	51.0
Current Cannabis use %		25.5	17.0
Antipsychotic medication %			
	No antipsychotic medication	14.6	2.0
	Loose binding	39.6	51.0
	Medium binding	10.4	24.5
	Tight binding	31.3	16.3
	Other or not registered	4.2	6.1
Characteristic symptoms			
	Disoriented symptoms	0	4
	Depressive symptoms	1	0
	Cakatone type symptoms	0	1
	Undifferentiated symptoms	4	7
	Paranoid symptoms	18	17
	Psychotic symptoms	8	9
	Schizophrenic affective symptoms	7	3
	Schizophrenic symptoms	3	3
Diagnosis according to DSM-IV-TR			
	Schizophrenia disorder	29	34
	Schizoaffective disorder	7	3
	Psychotic disorder NOS	10	9
	Other psychotic diagnosis	4	4
Clinical variables at Baseline			
PANSS Negative symptoms		17.77 (5.36)	17.87 (5.73)
PANSS positive symptoms		11.76 (3.39)	10.92 (3.46)
PANSS general symptoms		28.42(6.5)	27.06(5.7)
EQ-5D-5L		0.76 (0.15)	0.74 (0.2)

CBTsa: Cognitive behaviour therapy focusing on social activation; TAU: treatment as usual; PANSS: Positive and Negative Syndrome Scale for schizophrenia; EQ-5D-5L: EuroQol 5 dimensions 5 levels.

### 3.2 Loss to follow-up

Participation rate at 3 months was 81.6% in the CBTsa group (n = 40) and 78% (n = 39) in the TAU group. Thirty-nine patients in the CBTsa group, and 35 TAU patients still took part in the study at the 6-month follow-up. Drop-out was not associated with clinical or socioeconomic factors such as education, DSM IV diagnosis or antipsychotic medication use.

### 3.3 Clinical outcomes

Treatment response at 3 months directly post-treatment was 34.7% in the CBTsa condition and 22% in the TAU condition. At 6 months follow-up, the response rates had risen to 36.7% and 24.0%, respectively. Regarding QOL, patients in the CBTsa group cumulated a mean QALY gain of 0.40 over the six months follow-up whereas the TAU group gained 0.37 (see Table 2). A significant difference of 0.034 QALY was found in favour of the intervention group (b = 0.034, SE = 0.014, t = 2.420, p = 0.018). Regarding treatment response, no significant difference was found between both groups (OR = 1.839, SE = 0.817, z = 1.37, p = 0.170).

**Table 2:** Clinical outcomes and utilities at BS; FU3M; and FU6M per group.

		Intervention group (SD) (N = 49)			Treatment as usual (SD) (N = 50)		
		T0	T1	T2	T0	T1	T2
***Responder rate based on PANSS***		N/A	34.70	36.70	N/A	22.00	24.00
***Utilities / QOL***							
Utilities (NL-tariff)		0.75 (0.15)	0.82 (0.14)	0.83 (0.17)	0.72 (0.19)	0.71 (0.25)	0.79 (0.17)
	Total QALYs (NL-tariff)^a^			0.40			0.37
Utilities (UK-tariff)		0.81 (0.12)	0.87 (0.11)	0.87 (0.13)	0.79 (0.15)	0.79 (0.19)	0.85 (0.13)
	Total QALYs (UK-tariff)^a^			0.43			0.40

^a^ Total QALYs are calculated over 6 months (max QALY is 0.5)

SD: Standard deviation; T0: baseline; T1: follow-up at 3 months; T2: follow-up at 6 months; QOL: Quality of life

### 3.4 Costs

At baseline, productivity losses were higher in the CBTsa group (see S1 Table). Hence, bootstrapped SURE models were adjusted to correct for this baseline difference.

Average total costs per-patient (healthcare costs, patient & family costs, and productivity losses) at 6 months follow-up were €45 894 for the CBTsa group and €34 977 for the control group. The largest cost differences occurred in health care and stemmed from productivity losses. A more detailed overview on the cumulative costs over the follow-up is presented in Table 3.

**Table 3:** Cumulative costs over the 6 months follow-up period by condition (CBTsa vs TAU), in 2015 Euros.

	CBTsa group			TAU group		
		N = 49	95%CI		N = 49N = 50	95%CI
		Lower bound	Upper bound		Lower bound	Upper bound
**Intervention costs**	1 579.93			0.00		
**Health care costs**						
GP visits	93.50			60.00		
Specialist visits	548.62			530.39		
Nurse specialist	400.84			176.93		
Other visits**	949.19			678.63		
Inpatient stay	11 247.00			8 671.27		
Daycare	4 014.00			1 581.27		
AP medication	5 894.86			7 401.41		
Other medicaion	7.60			8.98		
Total health care costs*	*29 282*.*16*	*13 950*.*75*	*48 119*.*08*	*21 152*.*74*	*11 788*.*76*	*31 592*.*94*
**Patient & family costs**						
(Formal) Home care	14 287.14			11 501.05		
Informal care	36.86			142.95		
Special home care***	4 681.88			3 626.63		
Total patient & family costs*	*14 232*.*32*	*7 520*.*31*	*22 117*.*93*	*13660*.*59*	*5 837*.*63*	*22 895*.*63*
**Productivity losses**						
Absenteeism in paid work	418.32			14.64		
Presenteeism in paid work	222.26			15.05		
Unpaid work	119.48			93.37		
Total production losses*	*799*.*49*	*264*.*29*	*1523*.*04*	*318*.*61*	*97*.*39*	*256*.*41*
Total costs after 6 months*	45 893.91	29 675.57	67 049.98	34 976.60	23 505.07	48 263.83

* (Sub-)totals based on multiple imputation estimates

** Included paramedical care, dietitian, and alternative healing

*** Includes sheltered living and supervised living

CBTsa: Cognitive behavior therapy focusing on social activation; TAU: treatment as usual

### 3.5 Health-economic evaluation

**Fig 2:**
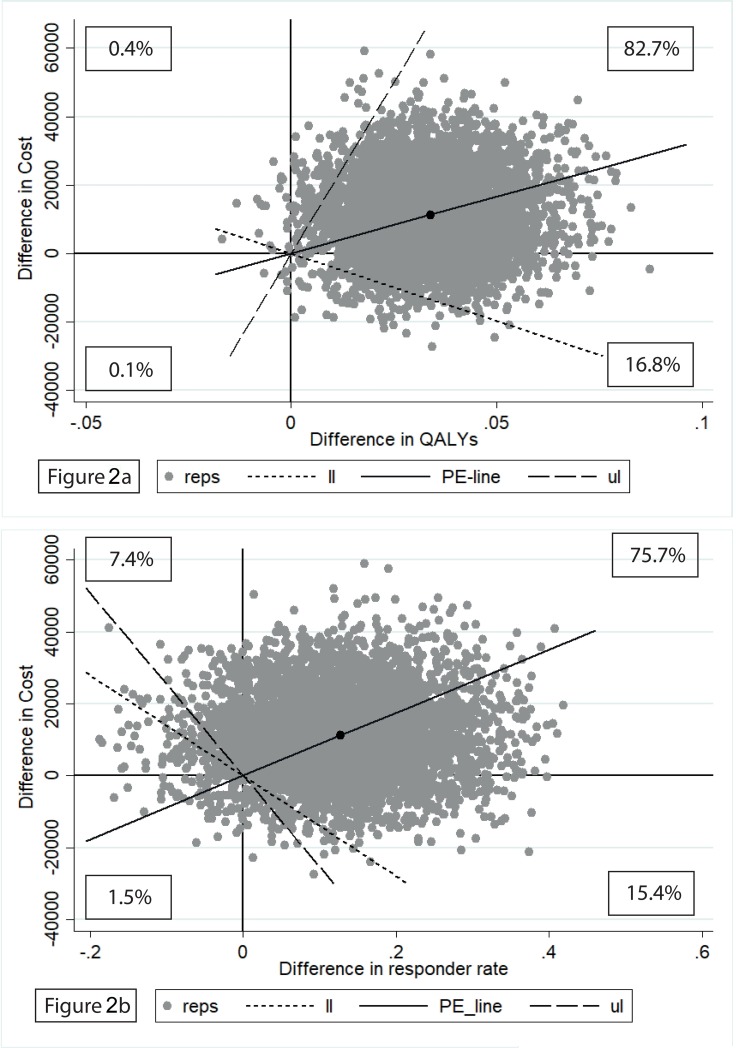
a) Cost-effectiveness plane of QALY scores at 6 months’ follow-up (costs per QALY gained); Fig 2B) Cost-effectiveness plane of responder rate (≥5 decrease in PANSS score) at 6 months’ follow-up (costs per extra responder). Percentages refer to the percentage of observations in respective quadrant; Reps: ICER replication; Ll: lower limit of the 95% confidence interval; PE: mean ICER; Ul: upper limit of 95% confidence interval.

**Fig 3:**
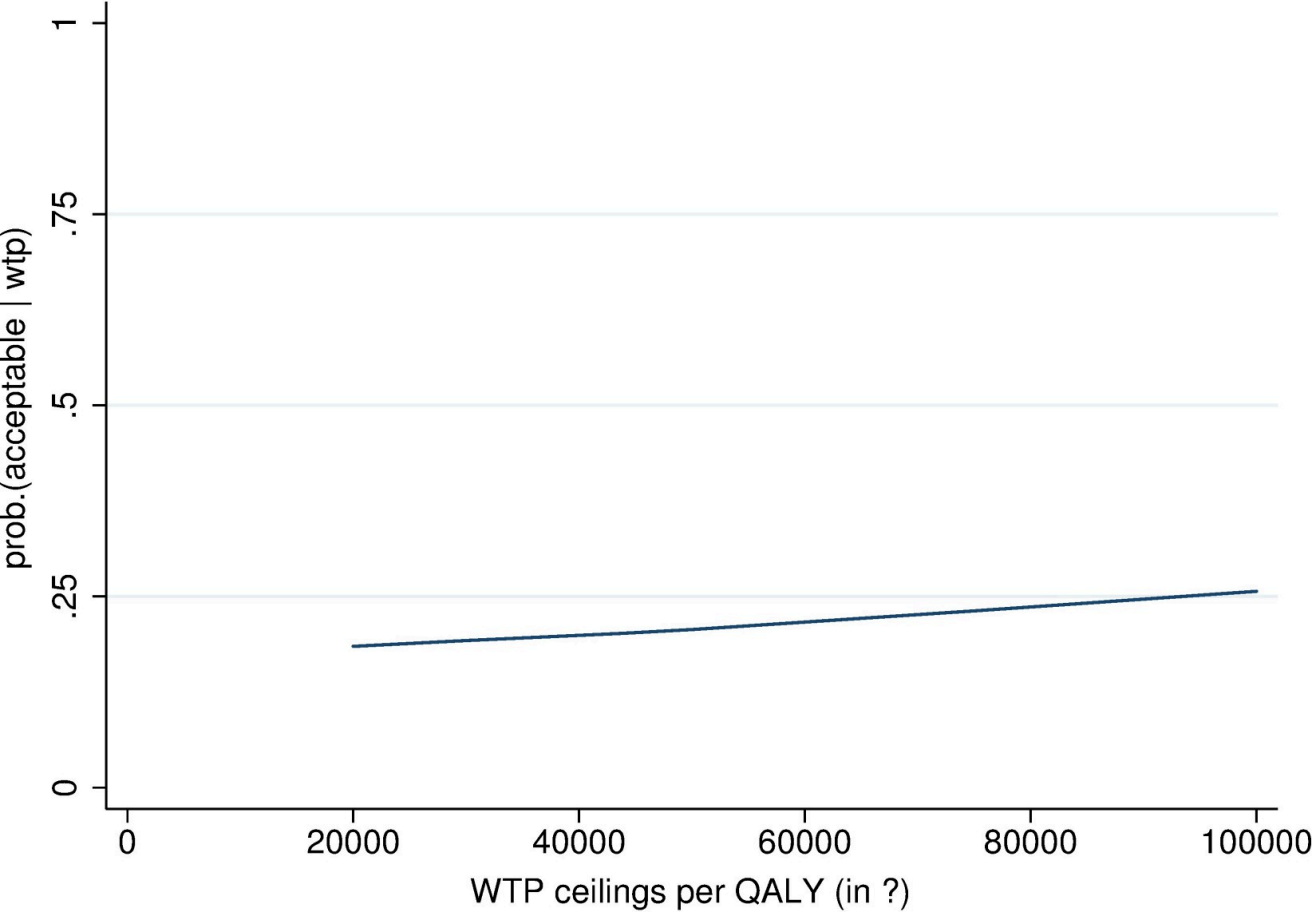
Cost-effectiveness acceptability curve of costs per QALY gained at 6 months.

The mean ICER was estimated at €87 886 per treatment responder. The mean ICUR was €428 842 per additional QALY gained. Results of the bootstrap replications are presented in Fig 2A and 2B. Fig 3 shows the probability that CBTsa is cost-effective given various willingness-to-pay (WTP) ceilings. From Fig 3, it can be concluded that if one would be willing to pay (a maximum of) €80 000 per QALY gained, the probability that the MCI would be cost-effective is only slightly higher than 25%.

Using INBRA, no subgroups were identified for which CBTsa was significantly more (or less) cost-effective. Looking at main effects, no differences were found between different participants of Dutch or non-Dutch decent (all p-values ≥ 0.093), social classes (all p-values ≥ 0.726), gender (p = 0.903), age (p = 0.092), or educational level (all p-values ≥ 0.662) for both a willingness to pay of €20,000 or €80 000 per QALY. In addition, predictors were examined for possible interactions with condition which did also not result in significant outcomes. The detailed outcomes of the INBRA analysis can be obtained from the first author.

### 3.6 Sensitivity analyses

Correcting for baseline differences regarding anti-psychotic medication use resulted in an ICUR of €333 310 per QALY gained and an ICER of €58 348 per responder. Performing analyses from the health care perspective rather than the societal perspective resulted in an ICUR of €649 040 per QALY and an ICER of €139 380 per responder.

## 4. Discussion

### 4.1 Main findings

This study examined the cost-effectiveness and cost-utility of CBT focusing on social activation from a societal perspective. QALYs were significantly higher in the CBTsa condition compared to the TAU condition as measured over the follow-up period of 6 months. It is interesting to see improvements in the measurement of QALYs since the EQ-5D-5L includes only a single item tapping into the mental aspects of quality of life. At six months, albeit not statistically significant, the treatment response rate (i.e. ≥ 5-point decrease on the PANSS negative symptom score) was higher in the CBTsa condition compared to the TAU condition. The current study demonstrated that CBTsa resulted in substantial additional costs per QALY gained. Looking at the WTP ceilings provided by the Council for Public Health and Health Care, i.e. €20 000 –€80 000 per QALY, the ICURs found in this study were about 3.7 times higher than its acceptable upper limit.

### 4.2 Strengths and limitations

This study is the first to evaluate the cost-effectiveness of CBT for social activation (CBTsa) in recent onset schizophrenia spectrum disorders. Such a study is important because the negative symptoms of these disorders (such as social inactivity) tend to linger on when left untreated and do much to compromise the QOL in these patients. Our study was therefore set out to evaluate the impact of add-on CBTsa on the participants’ negative symptoms, QOL and, in addition, to see if offering this add-on CBT intervention would be sustainable economically. Another strength of the study was that outcome assessment was conducted by psychologists blind to treatment allocation, but a double blind study was not possible due to the nature of this psychological intervention. Our study also suffered from a number of limitations and the findings of our study should be considered in light of these limitations.

First, both the experimental (CBTsa) and the TAU condition were directed at social activation, albeit in varying degrees. This is to say that the experimental contrast between the trial conditions was not very pronounced and may have led to a small effect in the outcome variables of interest (reduction in PANSS negative symptoms and improvement of health-related QOL). In this context it should be noted that in the Netherlands the standard treatment of psychosis and schizophrenia spectrum disorders is guideline congruent and well implemented. Hence, results of this study are not necessary generalizable to other countries with a less intensive or lower quality of standard care. On the other hand, the new add-on CBTsa was associated with a high dropout rate (of 50%) from that intervention, which may have reduced the experimental contrast even further with regard to both PANSS negative symptom severity and EQ-5D-5L health-related QOL [30]. In health economic evaluation generic QOL assessment, in particular the EQ-5D-5L, is recommended as an outcome variable, and as indicated we had a specific interest in evaluating the intervention’s impact on QOL. However, the EQ-5D has been questioned for its suitability for mental disorders, especially in the psychosocial domains of QOL [34]. Nonetheless, the EQ-5D-5L is still recommended as a generic (not disease-specific) instrument to assess QOL across all kinds of diseases and disorders [35].

Second, we were not successful in recruiting the required number of patients (n = 112), but were left with n = 99 (49 in the experimental and 50 in the control condition), rendering the study underpowered. The lack of power was further aggravated by a loss to follow-up (20% in the experimental condition and 30% in the control group). Nonetheless, all our analyses were conducted on an intention-to-treat basis with missing observations imputed.

Third, the follow-up of this study was conducted 6 months post baseline and 3 months post intervention, thus precluding the evaluation any longer-term treatment costs and effects.

Fourth, as is often the case in psychological intervention, blinding of patients for the intervention was not possible. Although measurements were carried out by trained psychologists or master students in clinical psychology who were all blind to treatment condition, our results could potentially be biased by the lack of blinding.

Finally, health care resource use was assessed using self-report, which may have caused recall bias to some extent. However, it is expected that this potential bias would be equal between both groups.

### 4.3 Conclusions

The lack of cost-effectiveness in our study may be partly explained by the already high quality of care provided to patients in standard practice, especially immediately after the first psychotic episode. The quality of care has much been boosted since the publication of the multidisciplinary guidelines for schizophrenia in the Netherlands [36]. In the treatment as usual condition much effort was directed at improving social functioning, and the findings from current study show that CBTsa does not provide an additional advantage in improving social withdrawal or quality of live compared to the well-developed and firmly implemented TAU. An alternative explanation for our null finding might be that CBTsa is not effective in the early phase of schizophrenia, because the negative consequences of social withdrawal may become manifest only in later disease stages. However, since social withdrawal occurs early in psychosis and the schizophrenia spectrum disorders and since persisting withdrawal predicts further withdrawal [37], we believe that this alternative explanation is unlikely. Hence, in this study CBT directed at social withdrawal appeared neither to result in less severe social withdrawal nor in an increase QOL when compared to TAU. The results of this study must, however, be interpreted with caution because the study was underpowered and could therefore not detect more subtle differences between the conditions. However, the magnitude of the differences in effect between conditions was not clinically relevant, and even when we would have been able to include more participants it seems unlikely that we would have seen clinically relevant benefits of the CBTsa condition over the TAU condition. In addition, as is often the case in trial-based economic evaluations, the study was powered neither for testing differences in QALYs nor costs, which is particularly relevant for the INBRA analysis.

To conclude, some positive trends were observed, but this underpowered study did not show statistically significant differences regarding negative symptoms and a small significant difference in QOL between both conditions. The lack of effectiveness was also reflected in the unfavourable cost-effectiveness ratios. Larger studies, preferably with other control conditions, are needed to determine to what extend CBT for social withdrawal may improve functional status and health-related QOL in newly diagnosed patients with schizophrenia spectrum disorders. Nonetheless, the current study does not support the hypothesis that a dedicated CBT approach focusing on social withdrawal has added value for patients in the early course of schizophrenia, at least not when compared to care as usual which is congruent with the current standards of care in the Netherlands.

## Ethical standards

The authors assert that all procedures contributing to this work comply with the ethical standards of the relevant national and institutional committees on human experimentation and with the Helsinki Declaration of 1975, as revised in 2008. The study was approved by the Medical Ethics Committee of the Academic Medical Center Amsterdam. The trial is registered at ClinicalTrials.gov registry under NCT03217955.

## Supporting information

S1 Table Average per-patient baseline costs for CBTsa and ST group (one month; in 2015 Euro). (DOCX)

S1 File Detailed research protocol. (PDF)

S2 File Completed CONSORT checklist. (DOC)
