# Supplementary material for: Economic evaluation of brief cognitive behavioural therapy for social activation in recent-onset psychosis
Source: PLoS One. 2018 Nov 12;13(11):e0206236. doi: 10.1371/journal.pone.0206236 (PMC6231612; doi:10.1371/journal.pone.0206236)
Supplement: S1 Table — (DOCX) [file pone.0206236.s001.docx]

S1 Table Average per-patient baseline costs for CBTsa and ST group (one month; in 2015 Euro)

|  | Intervention group | |  | ST group | |  |
| --- | --- | --- | --- | --- | --- | --- |
|  | N=49 |  | 95%CI | N=50 |  | 95%CI |
|  |  | Lower bound | Upper bound |  | Lower bound | Upper bound |
| Total health care costs | 10855.23 | 5884.62 | 17237.82 | 7746.54 | 2959.17 | 13591.85 |
| Total patient & family costs | 1425.99 | 599.96 | 2636.73 | 2551.03 | 1025.96 | 4334.39 |
| Total productivity losses | 173.76 | 13.75 | 393.99 | 6.44 | 0.00 | 14.82 |
