# Supplementary material for: Economic evaluation of brief cognitive behavioural therapy for social activation in recent-onset psychosis
Source: PLoS One. 2018 Nov 12;13(11):e0206236. doi: 10.1371/journal.pone.0206236 (PMC6231612; doi:10.1371/journal.pone.0206236)
Supplement: S1 File — (PDF) [file pone.0206236.s002.pdf]

SOFIA

Cognitive Behavioural Therapy – **S**ocial **F**unctioning **I**n **A**dolescents with recent onset schizophrenia

(Maart 2014)

PROTOCOL TITLE Cognitive Behavioural Therapy - Social Functioning in Adolescents in Adolescence with recent- onset schizophrenia

**Protocol ID**	**837001401**
**Short title**	**SOFIA**
**Version**	**1**
**Date**	**November 2013**
**Coordinating investigator/project leader**	**Eva Velthorst/ Carin Meijer**<br>**PA1-129**<br>**T: +31 (0)20 8913631**
**Principal investigator(s) (in Dutch: hoofdonderzoeker/ uitvoerder)**<br>***<Multicenter research: per site>***	**AMC, Amsterdam:**<br>**Prof. dr. L. de Haan**<br>**PA0-158**<br>**T: +31 (0)20 891 35 74**<br><br>**Centrum Eerste Psychose Parnassia, Den haag:**<br>**Drs. Winfried Laan**<br>**088-3575279**<br><br>**Altrecht ABC team, Utrecht:**<br>**Tonnie Staring**<br>**06 815 99 505**<br><br>**Arkin, Amsterdam:**<br>**Reinaud van de Fliert**<br>**020-4639386**
**Sponsor (in Dutch: verrichter/opdrachtgever)**	**Academic Medical Center, Amsterdam**
**Subsidising party**	**ZonMw**
**Independent expert (s)**	**Drs. E. Barkhof, psychiater**<br>**T: +31 (0)20 8913641**<br>**Email: e.barkhof@amc.uva.nl**

PROTOCOL SIGNATURE SHEET

Name	Signature	Date
For non-commercial research,<br>Head of department:<br>Prof. Dr. D. A. P. Denys<br><br>Kernhoogleraar Psychiatrie, Universiteit van Amsterdam		
Coordinating Investigator/Project leader/Principal Investigator:<br>Prof. dr. L. de Haan, psychiater		

TABLE OF CONTENTS

1. OBJECTIVES 9
2. STUDY DESIGN 10
STUDY POPULATION 12
2.1 Population (base) 12
2.2 Inclusion criteria 12
2.3 Exclusion criteria 12
2.4 Sample size calculation 12
3. TREATMENT OF SUBJECTS 14
3.1 Investigational product/treatment 14
3.2 Use of co-intervention (if applicable) 16
4. METHODS 17
4.1 Study parameters/endpoints 17
4.1.1 Main study parameter/endpoint 17
4.1.2 Secondary study parameters/endpoints (if applicable) 17
4.1.3 Other study parameters (if applicable) 17
4.2 Randomisation, blinding and treatment allocation 17
4.3 Study procedures 17
4.4 Withdrawal of individual subjects 20
4.5 Replacement of individual subjects after withdrawal 20
4.6 Follow-up of subjects withdrawn from treatment 20
4.7 Premature termination of the study 20
5. SAFETY REPORTING 21
5.1 Section 10 WMO event 21
5.2 AEs, SAEs and SUSARs 21
5.2.1 Adverse events (AEs) 21
5.3 Primary study parameter(s) 21
5.4 Secondary study parameter(s) 21
5.5 Other study parameters 24
6. ETHICAL CONSIDERATIONS 25
6.1 Regulation statement 25
6.2 Recruitment and consent 25
6.3 Benefits and risks assessment, group relatedness 25
6.4 Compensation for injury 26
6.5 Incentives (if applicable) 26
6.6 Handling and storage of data and documents 27
6.7 Amendments 27
6.8 Annual progress report 27
6.9 End of study report 27
6.10 Public disclosure and publication policy 27
7. REFERENCES 28

LIST OF ABBREVIATIONS AND RELEVANT DEFINITIONS

**ABR**	**ABR form, General Assessment and Registration form, is the application form that is required for submission to the accredited Ethics Committee (In Dutch, ABR = Algemene Beoordeling en Registratie)**
**AE**	**Adverse Event**
**CCMO**	**Central Committee on Research Involving Human Subjects; in Dutch: Centrale Commissie Mensgebonden Onderzoek**
**CV**	**Curriculum Vitae**
**EU**	**European Union**
**IC**	**Informed Consent**
**METC**	**Medical research ethics committee (MREC); in Dutch: medisch ethische toetsing commissie (METC)**
**Sponsor**	**The sponsor is the party that commissions the organisation or performance of the research, for example a pharmaceutical<br>company, academic hospital, scientific organisation or investigator. A party that provides funding for a study but does not commission it is not regarded as the sponsor, but referred to as a subsidising party.**
**WMO**	**Medical Research Involving Human Subjects Act (in Dutch: Wet Medisch-wetenschappelijk Onderzoek met Mensen**

SUMMARY

Rationale: There is growing consensus that targeting negative symptoms such as social withdrawal is essential to be able to preserve social participation, thereby reducing the high yearly costs of schizophrenia. Aaron T. Beck, founder of Cognitive Behavioural Therapy (CBT), and colleagues have developed and investigated a new CBT approach, in which they target inactivity in a chronic schizophrenia population with severe negative symptoms The therapy is based on accumulating evidence that dysfunctional beliefs in conjunction with neurocognitive impairments can impede social functioning. These results suggest that CBT can be highly successful in establishing clinically meaningful improvements. However, the therapy has not yet been investigated in a recent-onset population.

Objective: To evaluate the applicability and (cost-) effectiveness of a shortened, partly group based, Cognitive Behavioural Therapy focussing on social activation (CBTsa) in patients with recent onset schizophrenia.

Hypotheses: 1) We hypothesize that CBT focused on social activation (CBTsa) in a recent-onset population will result in a substantial reduction in severity of negative symptoms, in particular social withdrawal.

2) We expect that CBTsa will lead to an improvement in terms of Quality of Life and overall functioning.

3) We expect this intervention to result in a reduction in need for care and QALY gain as a consequence of improvement in symptoms and social functioning.

Study design: Single blind randomized controlled trial with 6 month-follow up.

Study population: Patients between 18 and 35 years old with negative symptoms of at least moderate severity, and who have been recently (< 2yrs) diagnosed with schizophrenia.

Intervention (if applicable): Individual and group-based CBT intervention targeting social withdrawal.

Main study parameters/endpoints: Change in negative symptoms, Social functioning, and quality of life, Productivity losses.

Nature and extent of the burden and risks associated with participation, benefit and group relatedness: <u>Burden</u>: Patients will undergo a 2-hour during structured interview and will carry along a mobile device with which participants are prompted by a beep at random intervals throughout the day (for a 6-day period) to report about their current experiences and withdrawal behaviour. These assessments will be repeated post-treatment and at 6-month follow-up. <u>Risks</u>: No risks are attached to this study. <u>Benefits</u>: With this intervention early in the course of the disorder we hope a) to prevent social withdrawal and diminish negative symptoms, thereby preventing the young patient from dropping-out of his/her social roles and from early social exclusion and/ or b) to help regain social roles when drop-out already occurred.

INTRODUCTION AND RATIONALE

Scientific rationale

Poor social functioning is a severely disabling characteristic of schizophrenia. It is more persistent over time than positive symptoms and despite antipsychotic treatment, functional prognosis has remained generally poor [1]. An important aspect of poor social functioning is the tendency to withdraw from social contact, one of the 'negative symptoms' of schizophrenia. There is growing consensus that targeting negative symptoms such as social withdrawal is essential to be able to preserve social participation [2]; thereby reducing the high yearly costs of schizophrenia.

Aaron T. Beck, founder of Cognitive Behavioural Therapy (CBT), and colleagues have developed and investigated a new CBT approach, in which they target inactivity in a chronic schizophrenia population with severe negative symptoms; a subgroup that has always believed to be highly treatment resistant. The therapy is based on accumulating evidence that dysfunctional beliefs in conjunction with neurocognitive impairments can impede functioning [3]. Its primary focus is to help patients overcome isolation and inactivity, thereby improve quality of life [2], rather than primarily focussing on the reduction of psychotic symptoms. Grant, Beck and colleagues [3] found that patients treated with CBT not only showed more improvement in global functioning than patients in the Standard Treatment condition, they also showed a greater reduction in avolition/apathy and psychotic symptoms. These results suggest that CBT can be highly successful in establishing clinically meaningful improvements.

Thus far, this intervention has only been investigated in patients with chronic schizophrenia although there is increasing evidence that social withdrawal is also prominent in the early phase of the illness. Moreover, there is growing consensus that early intervention is more effective in improving illness course than intervention at a later stage of the illness [4]. In the present study we aim to examine whether a relatively short, partly group based CBT targeting negative symptoms in recent-onset schizophrenia patients results in reduced social withdrawal, need for care and improvement in quality of life and in overall functioning compared to treatment as usual.

Clinical Relevance

We expect that intervening early is more effective in improving social integration than at a later stage when dysfunctional behaviours have become embedded in a fixed pattern of behaviour. We anticipate that this intervention will result in substantial gains regarding negative symptoms, and in particular social withdrawal. Intervening in this early phase of the illness is expected to a) prevent the young patient from dropping-out of his/her social roles

and from early social exclusion and/ or b) to help regain social roles when drop-out already occurred.

Anticipated Cost-Effectiveness/ Budget Impact

A secondary aim of the present study is to examine the cost-effectiveness of this new type of intervention. We based our estimated cost-effectiveness on the following calculation:

1. Costs of the intervention per patient are estimated at 800 Euro.
2. We expect a reduction in negative symptoms with an effect size of .66 compared to standard care only [3].
3. There is a small effect or no effect of usual care on negative symptoms [3&5]. Since the exact effect is unclear and our concern is the additional effect, we set the effect of usual care at 0, and use the effect of our intervention (effect size 0,66) compared to usual care. Assuming that negative symptoms resemble depression, and that negative symptoms persist half a year, this amounts to a QALY gain of 0,66 (effect size)*0,172 (conversion factor d->dw)*0,5 (duration of half a year) per patient= 0.057.
4. The costs for an additional QALY then are 800/0,057 = €14.035,09 per additional QALY.
5. This is below the €20.000 cut-off, as a result of which the intervention may be considered costeffective

Existing evidence of effectiveness

In a recently finished pilot study of our research group [6], 21 patients (of which 4 recent-onset) were treated in a RCT trial based on the principles of the Grant et al. [3] study. The researchers concluded the following:

(1) Feasibility of the treatment and treatment manual was good,

(2) Positive effects: Intention-to-treat analyses showed a within group effect size of 1.26 on negative symptoms (t=6.16, | sig=.000); and

(3) the recent-onset patients showed a greater reduction in negative symptoms than the chronic schizophrenia population.

Innovative character

To the best of our knowledge, no other study has examined the applicability of a concise version of the CBT as developed by Grant et al in a recent-onset population. Notwithstanding the highlighted advantages of the CBT intervention in chronic schizophrenia, a main drawback is its rather long duration (18 months on a weekly basis), bringing along substantial costs. We have developed a shorter, partly group-based intervention, adapted to the specific needs of this young group in the early stage of the illness. We expect this adapted approach to be more feasible and cost-effective compared to the original intervention.

1. OBJECTIVES

Primary Objective:

The primary aim of this project is to examine a shortened (+/- 20 sessions) and partly group-based version of the new and promising CBT approach tested in a chronic population (from now: 'Cognitive Behavioural Therapy – Social Activation' (CBTsa)), for its applicability and effectiveness in a recent-onset population. The CBTsa will be adjusted to the specific needs of the young recent-onset cohort.

Secondary Objective(s):

We secondly aim to explore the cost-effectiveness of this new intervention (i.e. the balance between costs and health outcomes for CBTsa compared with treatment as usual(ST).

Hypotheses:

1) We hypothesize that CBT focused on social activation (CBTsa) will result in a substantial reduction in severity of negative symptoms, and in particular social withdrawal.

2) We expect this intervention to result in a reduction in need for care and QALY gain as a consequence of improvement in symptoms and social functioning.

3) We expect that CBTsa will lead to an improvement in terms of Quality of Life and overall functioning and symptomatology.

2. STUDY DESIGN

Study Design

The present study entails a single-blind two-level RCT directed at patients with recent onset schizophrenia (see 'flowchart'). Participants will be randomly assigned to intervention condition (Cognitive Behavioral Therapy- Social Activation (CBTsa) plus
Standard Treatment (ST)), or to the control condition (ST alone). Patients will be stratified by sex, as females with recent onset schizophrenia have a better prognosis and may respond differentially to CBTsa. A baseline test battery will be employed (see below) to examine causes of social withdrawal and interaction with treatment outcome. Follow-up assessments (repetition test battery) will take place directly after the intervention period and 6 months post-treatment.

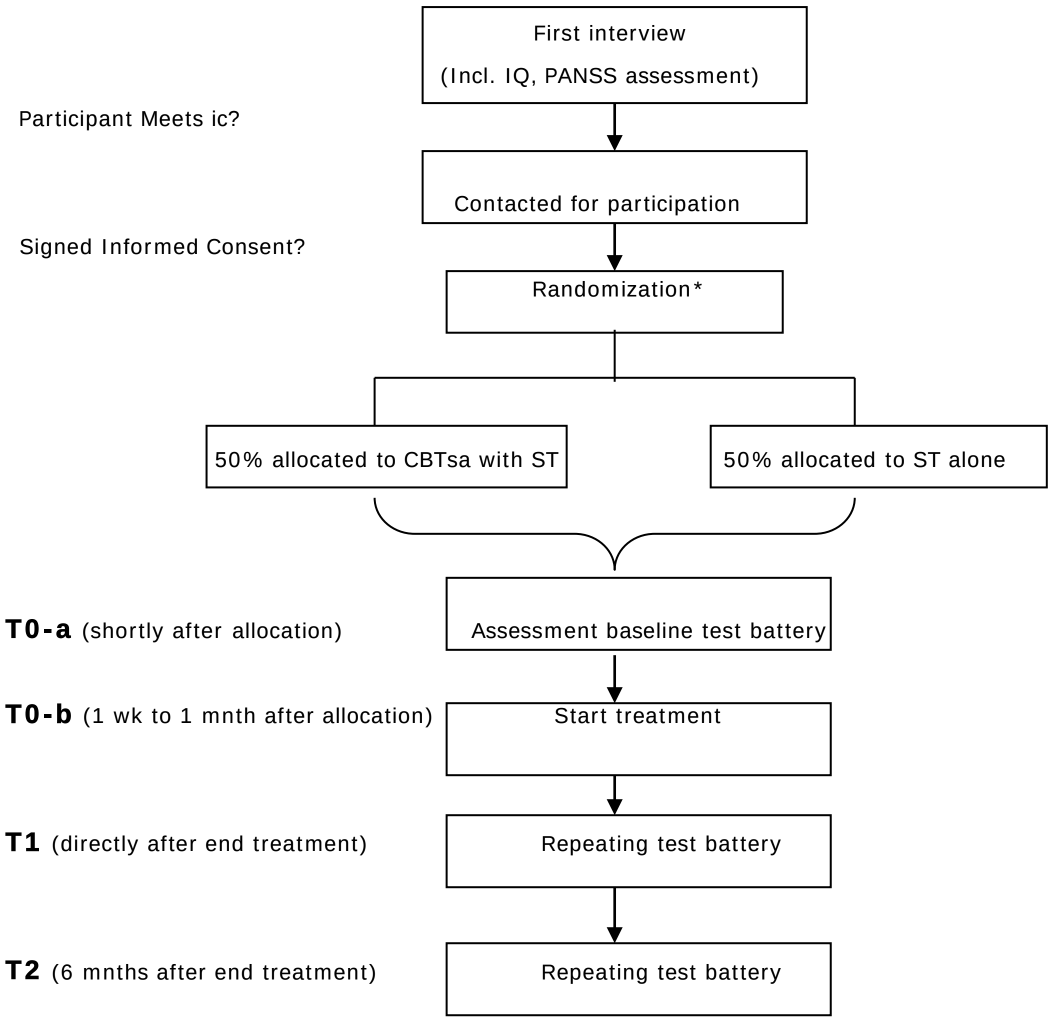

Figure 2. Flowchart of the phases of the randomized trial for the two groups. CBTsa indicates cognitive behavioural therapy – social activation; ST = standard treatment.

* EV will conduct the baseline- and follow- up assessments and will be kept blind of the study condition.

Study population/ datasources

Study sample: patients hospitalized or attending day-treatment or receiving outpatient care at the Department of Early Psychosis, Amsterdam, Early Intervention Psychosis service of Arkin, Amsterdam (Vroege Interventie Psychose: VIP) the psychosis department of the ABC team, Utrecht and Centrum First Psychosis, Parnassia, The Hague. To maximise engagement, group therapy will be incorporated in the day program. Individual therapy sessions will be delivered flexibly (time, location). All participants are embedded within a low-threshold intensive outreaching care system (aligned with our academic department).

STUDY POPULATION

2.1 Population (base)

Study sample: patients hospitalized or attending day-treatment or receiving outpatient care at the Department of Early Psychosis, Amsterdam, Early Intervention Psychosis service of Arkin, Amsterdam (Vroege Interventie Psychose: VIP) the psychosis department of the ABC team, Utrecht and Centrum First Psychosis, Parnassia, The Hague. To maximise engagement, group therapy will be presented as part of the standard dayprogram. Individual therapy sessions will be delivered flexibly (time, location). All participants are embedded within a low-threshold intensive outreaching care system (aligned with our academic department).

2.2 Inclusion criteria

In order to be eligible to participate in this study, a subject must meet all of the following criteria:

(1) recent onset schizophrenia or related disorder (start antipsychotic medication <2 yr);

(2) Social withdrawal (> 3 moderate severity on the PANSS N4; Passive/apathic social withdrawal; range 0-7);

(3) Aged 18-35 years;

(4) Fluent in Dutch

(5) IQ>70;

(6) Able and willing to give informed consent

2.3 Exclusion criteria

A potential subject who meets any of the following criteria will be excluded from participation in this study:

(1) Younger than eighteen years of age;

(2) No mastery of the Dutch language;

(3) Negative symptoms as a consequence of positive symptoms (e.g. withdrawal due to paranoid delusions). Positive symptoms as such are not an exclusion criterion; only when they are considered to be the primary cause of the negative symptoms, in which case CBT focused on positive symptoms or another type of intervention for positive symptoms is called for.

2.4 Sample size calculation

The effectsize to calculate our estimated sample size is based upon a recent groundbreaking study of Grant and colleagues (one of our co-applicants), who carried out an intervention with

similar ingredients as ours in a more chronic schizophrenia population. In their study, also specifically targeted to reduce negative symptoms and to socially activate their patient sample, they found that patients receiving cognitive therapy showed a greater mean reduction in avolition-apathy (adjusted mean [SE], 1.66 [0.31] vs 2.81 [0.34], respectively; P=.01; between-group d=-0.66) as compared with those receiving standard
treatment. This outcome measure is comparable to the 'social withdrawal' outcome measure used in our study. Based on this results, and upon the commonly hold assumption that recent onset subjects are more likely to change than the more chronic population, we estimated to find an effectsize of (at least) .66 in our study as well. With an alpha of 0.5; power=80%; effect-size .66 [3], this would mean we need 36 participants per group to detect a true treatment difference. Taking into account an expected dropout rate of 20%, we would need to include 72 + 20% = 87 participants = 44 per group.

To take into account the ‘variance inflation’ factor (due to our multi-center design), we calculated the Intra Class Correlation Coefficient of the PANSS negative symptom scores of a study targeting a similar population and setting (GROUP), in which 4 centers participated. The ICC in this study was .146. Following the literature, we then used the following formula:
1 + (m- 1) x ICC (m= number of participating centers).

For our study this means we have to increase the sample size (87) by a factor of:
1 + (3-1) x.146 = 1.292

This results in a total estimated sample size of: 1.292 * 87= 112 individuals (56 per condition)

3. TREATMENT OF SUBJECTS

3.1 Investigational product/treatment

Intervention

The CBTsa therapy is based on accumulating evidence that dysfunctional beliefs in conjunction with neurocognitive impairments can impede functioning [3]. The core assumption of this therapy is that modifying dysfunctional beliefs will lead to increased engagement in constructive social activity in individuals with prominent negative symptoms [7]. Its primary focus is to help participants overcome isolation and inactivity, and thereby improve quality of life [2], rather than focusing primarily on reduction of psychotic symptoms.
CBTsa will target several defeatist beliefs and defeatist performance attitudes (e.g. 'if you cannot do something well, there is little point in doing it at all'). Apart from defeatist beliefs focusing on personal skills (such as cognitive functioning), negative expectancies regarding one's ability to experience pleasure (low anticipatory pleasure: 'I will not be able to enjoy this') are often reported by patients with schizophrenia. These types of beliefs combined with asocial beliefs (e.g. 'having close friends is not as important as most people say') have been found to result in (social) withdrawal and inactivity.

In the current intervention, Cognitive behavioural techniques will focus primarily on correcting these dysfunctional beliefs about pleasure, cognitive abilities, performance and social functioning. The intervention will consist of a group-based and an individual part, combining advantages of group processes and peer interaction with individual CBT. One major component of the group-based part is peer support (sharing experiences & practicing skills together with peers). The individual CBT sessions will be composed of personal case formulations, investigation of specific dysfunctional beliefs and behavioural experiments will be adressed.

In short, the intervention will constitute of:

1. Group sessions for 4 weeks, two sessions per week, 60 minutes per session, two trainers (a CBT therapist and a CBT assistant); eight participants, and;
2. Individual sessions (crystallizing learned skills, focus on individual needs) during 6-8 weeks, one session per week, 45 minutes per session.

The Group sessions (8 in total) will consist of:
Psycho-education, peer support, buddy-forming, social goal setting, breaking goals down into steps and planning them, find obstacles and dysfunctional cognitions, behavioural experiments, and imagery for executing goal steps.

The Individual Sessions will contain: Individual case formulations (including the person's main dysfunctional beliefs and associated behaviours), continuation of working on social goals and countering obstacles to these goals. Also, psycho-education about symptoms, the role of cognition / beliefs and consequences of current behaviour will be addressed. Specific cognitive techniques that will be used to investigate dysfunctional beliefs and behaviour are a.o. ‘Socratic dialogue’, behavioural and cognitive experiments; dimensional evaluation of negative or stereotype self image etc); cognitive imagery techniques (imagening the steps needed to achieve new goals); behavioural techniques (activity scheduling, exposure to new situations that trigger anxiety etc.). Finally family members / important others will be involved. The choice for a particular combination of the above mentioned techniques will be based on the individual needs and goals of the patients.

To ensure treatment engagement and adherence, sessions will be adapted to the particular group under study: young patients recovering from recent episode psychosis. Adaptations for specific subgroups of patients may include specific illness characteristics (for example cognitive problems; concentration), gender and ethnic background. For example, sessions will be kept as low-level as possible and adapted to specific interests of the adolescent group: attention will be paid to sharing experiences, guaranteeing an open and safe treatment atmosphere; 'fun' parts (such as social games) will be included in the program. Further, treatment session time and duration will be adapted to the needs of individual patients (for example shorter en more frequent sessions). We will strive to address diversity (regarding age, gender, ethnic background etc) in the group- based part of the intervention as well as the individual part, for example by involving family members or important others to discuss implementation of treatment gains in patient's daily life.

Standard treatment (ST)

Participants in both study conditions will receive ST. Participants are hospitalized or attending day-treatment at the Department of Early Psychosis, Amsterdam, the psychosis department of the ABC team, Utrecht, Parnassia Den Haag and collaborating (local community) mental health centers.

At minimum, Standard Treatment consists of antipsychotic medication and supportive therapy. Additional components of standard care for patients with psychotic disorders are psycho education, family support, physical health care, psycho motor therapy and vocational therapy.

3.2 Use of co-intervention (if applicable)

All participants are allowed to use antipsychotic medication, as part of their ST.

4. METHODS

4.1 Study parameters/endpoints

4.1.1 Main study parameter/endpoint

For comparison reasons and in congruence with earlier work (Velthorst et al., available on request), our primary outcome measure will constitute of a negative symptom sum score (see 5.3. study procedure). In addition, we will look separately at one of the specific impact of our intervention on withdrawal behaviour.

4.1.2 Secondary study parameters/endpoints (if applicable)

Quality of Life, Global Functioning, Productivity losses, Severity of symptomatology, Need for Care

4.1.3 Other study parameters (if applicable)

Substance Use, (socio-) demographic characteristics

4.2 Randomisation, blinding and treatment allocation

Randomisation shall take place after the patient has found to be eligible for the study and has given Informed Consent. Randomisation will take place within each participating center. We will make use of the digital randomiser www.randomiser.org. Randomisation will be coordinated by AF, a staff member of the psychosis department who is not involved in the research team and not familiar with both the assessments or intervention procedure. The results of the randomization will be concealed from the assessors. We will make every effort to keep assessors blind to treatment condition. The following strategies will be used to achieve blind ratings: research workers will not be involved in the randomization process, therapists and research workers will make use of different (secured) agendas, and work locations / rooms in order tot minimize the chance of potential blind breaks; patients will be frequently reminded by assessors not to talk about treatment allocation. Also, we will assess cases and study phase in which blindness breaks may appear. In those cases another research worker will perform subsequent assessments'.

Study procedures

After the participants have read and signed informed consent, and before treatment allocation, all patients will undergo a two hour during test battery carried out by an academic research assistant. Afterwards, participants will be asked to carry along an electronic device/ with an application with which participants are prompted by a beep at random intervals throughout the day (for a 6-day period) to report about their current experiences and withdrawal behaviour (see Experience Sampling Method (ESM; 9) table 1). The test battery will be repeated at the end of treatment, and 6-months post- treatment.

Patients allocated to the CBTsa condition will receive group sessions for 4 weeks, and 6-8 weeks weekly individual CBT sessions (see above).

Table 1. Test battery

**Primary outcome**	**Instruments**	**Characteristics Instrument**
Negative symptoms	Negative symptom domain of the Positive and Negative Syndrome Scale (PANSS; Kay et al., 1986)	Consists of 7 item clusters and produces a total score ranging from 7 (minimum) to 49 (maximum).
	Brief Negative Symptom Scale [BNSS; 10]:	Assesses objective and self-perceived negative symptoms and to be used in clinical trials [11]. 13 items that can be rated in a 15-minute interview. In contrast to other scales, the scale includes separate items for internal experiences and outward behaviour for asociality amongst others. Reliability analyses indicate that the BNSS has excellent internal consistency and temporal stability [11]. This scale has recently been officially translated by our research group by means of the back-forward translation method (article in progress).
	Experience Sampling Method (ESM [9]) currently transformed into a smartphone device:	Structured self-assessment technique ESM will be used to examine frequency of real-life activity and situational, cognitive, affective and behavioural aspects that precipitate social withdrawal. In contrast to the commonly used questionnaires, ESM not only yield more ecologically valid measures of social behaviour; it is also more attractive for the young target group, increasing the feasibility of the study.
**Secondary Outcome**		
Quality of Life	EQ-5D-5L; 12	To assess (changes in) quality of life we will make use of the EuroQol (EQ-5D-5L).
Global functioning	Global Assessment of Functioning Scale (GAF [13]).	This scale is rated on a scale from 1 to 100, for the current situation and for highest level in past year. 1 to 10, for example,<br>signifies ‘a persistent danger of severely hurting self or others (e.g., recurrent violence) OR persistent inability to maintain<br>minimal personal hygiene OR serious suicidal act with clear expectation of death’, whereas 91 to 100 on the other hand, stands

		for 'Superior functioning in a wide range of activities.., no symptoms.'
Productivity losses	SF-HLQ [14]	Effects on ability to perform paid and unpaid work
Positive and General Symptomatology	PANSS [8]	The PANSS is currently the most widely used scale to assess the severity of a variety of symptoms in patients with schizophrenia. Originally the PANSS consists of three sub scales: positive syndrome scale (item P1–P7), a negative syndrome scale (items N1–N7) and general psychopathology scale (item G1–G16).
Depression	Calgary Depression Scale for Schizophrenia (CDSS [15])	Nine-item scale specifically designed for schizophrenic patients that permits evaluation of depression independently from extrapyramidal and negative symptoms [15].
Inhibition/ Activation	Behavioural Inhibition/ Behavioural Activation Scales (BISBAS [16])	a 20-item self-rating questionnaire with good psychometric properties. Comprises 7 BIS items and 13 BAS items. Each item has 4 response options ranging from 1 (strongly agree) to 4 (strongly disagree). Aims to examine individual differences in the sensitivity for one of the two motivational systems that underly behaviour: A behavioral approach system (BAS) is believed to regulate appetitive motives, in which the goal is to move toward something desired. A behavioral avoidance (or inhibition) system (BIS) is said to regulate aversive motives, in which the goal is to move away from something unpleasant.
Need for care	Camberwell Assessment of Need (CAN) [17].	Includes 22 items (e.g. daytime activities, psychotic symptoms). All CAN items can be scored 0 (no problem), 1 (there was a problem, but the problem is met), 2 (unmet need) with a reference period including the last 3 months. The instrument is designed to measure all domains of need, a high level of internal consistency cannot be expected, but reliability has been reported ''acceptable'.
**Possible confounders**		
Substance use	Cannabis Experience Questionnaire (www.eu-gei.eu)	Assessing (experiences caused by) all kinds of drug use in detail (DiForti and colleagues, developed for the multi-centered EU-Gene-Environment Interaction study,
Socio-demographics	Socio-Demographic Schedule (www.eu-gei.eu)	Measure designed for EU-GEI to assess socio demographic characteristics

4.3 Withdrawal of individual subjects

Subjects can leave the study at any time for any reason if they wish to do so without any consequences. The investigator can decide to withdraw a subject from the study for urgent medical reasons.

4.4 Replacement of individual subjects after withdrawal

Dropout has been factored in at the sample size calculation. As we start treatment at group-level, individual subjects will not be replaced after withdrawal.

4.5 Follow-up of subjects withdrawn from treatment

When a subjects withdraws from the study, reasons for drop-out will be examined. The participant will be asked to participate in follow-up assessments without taking part in the treatment part of the study. Adequate clinical care will be delivered as needed.

4.6 Premature termination of the study

n case of detrimental effects as observed on group-level by RA's, clinicians or caregivers, the study will be terminated and adequate care will be provided.

5. SAFETY REPORTING

5.1 Section 10 WMO event

In accordance to section 10, subsection 1, of the WMO, the investigator will inform the subjects and the reviewing accredited METC if anything occurs, on the basis of which it appears that the disadvantages of participation may be significantly greater than was foreseen in the research proposal. The study will be suspended pending further review by the accredited METC, except insofar as suspension would jeopardise the subjects' health. The investigator will take care that all subjects are kept informed.

5.2 AEs, SAEs and SUSARs

5.2.1 Adverse events (AEs)

Adverse events are defined as any undesirable experience occurring to a subject during the study, whether or not considered related to [the investigational product / the experimental interventiont]. All adverse events reported spontaneously by the subject or observed by the investigator or his staff will be recorded.

STATISTICAL ANALYSIS

5.3 Primary study parameter(s)

Our primary and secondary study parameters are all of quantative and continuous nature. Evaluation of randomisation procedure and differential drop-out will be analysed by means of independent samples T-tests. We will apply mixed model repeated measures analyses to examine the effects of the intervention on negative symptoms over time, and the differential effect of the CBTsa condition relative to ST. In addition, we will make use of LISREL models to correct for attenuation due to measurement error.

5.4 Secondary study parameter(s)

- In equivalent analyses as mentioned above (*6.4. primary study parameters*), we will examine the effect of the intervention on the secondary study parameters (i.e. Quality of Life, general and positive symptomatology, depression, inhibition/ activation, need for care and overall functioning.

- Cost effectiveness and Budget impact Analyses:

<u>Cost Effectiveness Analysis (CEA):</u>

The economic evaluation will be conducted as a cost-effectiveness analysis, CEA, with costs / treatment responder, and a cost-utility analysis, CUA, using quality adjusted life years (EQ-

5D/QALYs) as a generic measure of health gains. Both the CEA and the CUA will be conducted from the societal perspective and in agreement with the intention to treat principle. The trial's follow-up measurements do not exceed the time horizon of one year and therefore neither costs nor effects will be discounted. Sensitivity analyses will be directed at uncertainty in the main cost-drivers.

Cost analysis

We will consider four types of costs: (1) the costs of offering the intervention, (2) costs stemming from health care uptake including the costs of medication, (3) patients' out-of-pocket costs, (4) costs stemming from productivity losses due to absenteeism and lesser efficiency while at work and owing to changes in the contractual number of work hours per week. The first two types of costs are also known as the direct medical costs and these will be based on the full economic costs of offering the interventions. For this we shall make use of the pertinent Dutch guideline for economic evaluation [18], and rely on the standard cost prices reported therein. The patients' out-of-pocket costs are known as direct non-medical costs and encompass the patients' costs of travelling to health services and parking costs incurred in the context of health care uptake. Finally, productivity losses will based on the gender and age specific friction costs, as outlined in the Dutch guideline for costing. Data on resource use (health care uptake) and productivity losses will be collected with the latest version of the Trimbos/iMTA Questionnaire Costs associated with Psychiatric illness, TiC-P, [19]. The TiC-P is the most widely used health service receipt interview for economic evaluations in the Netherlands.

CEA and CUA

The economic evaluation will be conducted alongside the randomised trial. The central clinical end-term will be treatment response, defined as an increase on the PANSS negative symptom score (with a 5-point increase meaning a clinical relevant change), for the CEA. For the CUA, the Dutch tariffs (utility weights) of the EQ-5D will be used for computing the QALYs [20]. The incremental cost-effectiveness ratio (ICER) will be computed to obtain the costs per treatment response and the costs per QALY gained. Stochastic uncertainty will be handled using 2,500 non-parametric bootstraps and plotting the simulated ICERs on the ICER plane. For decision-making purposes, the ICER acceptability curve will be plotted for various willingness-to-pay (WTP) ceilings for making judgements whether the adjuvant intervention offers good value for money relative to routine medical care alone. One-way sensitivity analyses directed at uncertainty in the main cost drivers will be performed to gauge the robustness of our findings across a range of likely values of those parameters.

Budget Impact Analysis

The budget impact analysis (BIA) will be conducted as outlined in Mauskopf [21] to assess how health care budgets are changed when offering adjuvant CBTsa over a range of implementation levels. The BIA will be conducted from various perspectives: (1) wider societal perspective, i.e. including productivity losses; (2) the more narrow perspective of the public purse (in Dutch: Budgettair Kader Zorg); down to (3) the narrow perspective of the health care insurer. In each perspective the following scenarios will be calculated a) a scenario in which the intervention is offered to 60% and 80% of the target group, and b) an extreme scenario in which 100% of the target group will be receiving the intervention. All scenarios will be compared with a base-case scenario, reflecting current care, where 0% of the target group is offered CBTsa.

Cost analysis

When taking the societal perspective, we shall consider the costs of offering the health care interventions (also the interventions offered in routine medical care for this particular target group), patients' out of pocket costs, and costs stemming from productivity losses. Treatment costs will be based on the full economic cost prices (standard cost prices as reported in the Dutch Costing Manual); productivity losses will be valued using the average gender and age specific productivity levels in the Dutch working population. When taking the perspectives of the public purse (BKZ) and the health insurance companies' perspective, then the focus will be restricted solely to the direct medical costs and for these costs use will be made of the average tariffs of the Dutch Health Authority (NZa).

Modelling approach

Use will be made of a health economic (Markov cohort) simulation model to be based on modelling techniques outlined in Briggs et al. [22]. The model will compare two health care systems: a base-case scenario representing usual care for patients and an alternative scenario representing usual care augmented by adjuvant CBTsa. Costs and effects will be modelled out over the short term (12 months) and longer-term (36 months). Long-term costs and effects will be discounted according to the Dutch guidelines. The model will be designed to conduct extensive sensitivity analyses over all cost, effect and discounting parameters simultaneously (cf. [22]). It is worth noting that the model will be populated using the excellent data available at AMC about the disease stages as occurring in the natural course of schizophrenia in a population at ultra-high risk of imminent psychosis, with further data on average PANSS scores per health state and the transition rates between those health states (cf. [23]). Therefore, we expect to be able to make a health economic model that will offer value beyond this study alone.

PATIENT OUTCOME ANALYIS (INCREMENTAL NET-BENEFIT REGRESSION ANALYSIS)

The intervention might be effective and cost-effective in some population segments, but not in others. For example, smaller treatment benefits might be expected in patients presenting with higher levels of paranoia, comorbid disorders (especially axis-2 disorders) and in ethnic minorities. Effect modification analysis is often used to shed light on heterogeneity in treatment response owing to target group diversity. In the context of health-economic evaluations, the same kind of information is obtained using incremental net-benefit regression analysis (INBRA).

Patient outcome analysis (INBRA)

INBRA relies on the same data as CEA, but now we prefer to have a clinical outcome on a continuous measurement scale [24]. For this we will make use of the PANSS. INBRA is essentially a regression analysis were the treatment dummy, a prognostically relevant population characteristic (e.g. Dutch vs non-Dutch ethnicity) and their interaction are regressed on net-benefits. Net-benefits, NB, are defined as, NB = (E * λ) – C, where E are effects (changes on the PANSS), λ is an unknown quantity representing the willingness to pay (WTP) for one unit of health gain (decrease on the PANSS-negative)
and C are the additional costs of offering the adjuvant CBTsa intervention. Because λ is an unknown quantity, we will use a range of plausible WTP levels in our analysis. Within the regression framework, INBRA essentially evaluates the net-benefits as a function of the effect modification and thus helps to answer the question: who benefits most? – or more precisely, are there any subgroups in which the intervention is particularly cost-effective. This information may aid future improvements of the CBTsa intervention such that it may become more cost-effective in subgroups that now do not fully benefit from the intervention or else these data may guide decisions about referring those patients to the intervention such that these people may derive full benefit from the intervention in a cost-effective way.

5.5 Other study parameters

Substance abuse, and socio-demographic variables (e.g. age, gender, medication prescription) will be added to the model as possible confounders.

6. ETHICAL CONSIDERATIONS

6.1 Regulation statement

The study will be conducted according to the principles of the Declaration of Helsinki (October 2008) and in accordance with the Medical Research Involving Human Subjects Act (WMO).

6.2 Recruitment and consent

Patients hospitalized or attending day-treatment at the Department of Early Psychosis, Amsterdam, the psychosis department of the ABC team, Utrecht and Centrum First Psychosis, Parnassia, The Hague will be checked for eligibility and asked for consent. To maximise engagement, group therapy will be presented as part of the standard dayprogram.

If a psychiatrist or physician of any the above-mentioned institutes sees a patient who meets our inclusion criteria, s/he will introduce the study, and intervention briefly to the patient. The academic research assistant will then approach the patient. To this end, s/he will provide oral and written information about the study (see Informed Consent).

In order to enhance the awareness of the study among professionals employed by the above institutes, the principal investigators and researchers will give talks at these institutes and stick posters to walls. The patients will be given approximately 48 hours to consider their participation.

Baseline assessments will be conducted by a RA blind to study condition and shortly after randomisation (see flowchart). The group therapy will commence after 16 participants (8 ST and 8 RCT) signed written informed consent; at maximum one month after baseline assessment.

6.3 Benefits and risks assessment, group relatedness

Risks: In the literature covering Cognitive Behavioural Therapy no risks, side effects or adverse effects have been described.

Benefits: recent evidence shows that modifying dysfunctional beliefs may improve negative symptoms and thereby functional outcome and social participation. With this intervention early in the course of the disorder we hope a) to prevent social withdrawal and diminish negative symptoms, thereby preventing the young patient from dropping-out of his/her social roles and from early social exclusion and/ or b) to help regain social roles when drop-out already occurred.

6.4 Compensation for injury

Given that no relevant risks are attached to the participation in the study, the Medical Ethical Committee obtained a release from the obligation to conclude special indemnity insurance for participants.

6.5 Incentives (if applicable)

Travel expenses and a total amount of 40 euro for the assessment of the test battery at baseline- end -of-treatment and 6 month follow-up will be reimbursed. Patients will receive 10 euros for completion of the first assessment, 20 if they participate in both baseline and the end-of-treatment assessment and 40 if they complete all three measures.

ADMINISTRATIVE ASPECTS, MONITORING AND PUBLICATION

6.6 Handling and storage of data and documents

Data will be handled confidentially anonymously. The handling of personal data will comply with the Dutch Personal Data Protection Act.

Data will be coded by participant number and connected to source data by a coordinating researcher (EV). Lieuwe de Haan (prinicipal investigator), Eva Velthorst and Carin Meijer (coordinating researchers) will have key to the code. Research data will be kept for 15 years.

6.7 Amendments

Amendments are changes made to the research after a favourable opinion by the accredited METC has been given. All amendments will be notified to the METC that gave a favourable opinion.

6.8 Annual progress report

The sponsor/investigator will submit a summary of the progress of the trial to the accredited METC once a year. Information will be provided on the date of inclusion of the first subject, numbers of subjects included and numbers of subjects that have completed the trial, serious adverse events/ serious adverse reactions, other problems, and amendments.

6.9 End of study report

The investigator will notify the accredited METC of the end of the study within a period of 8 weeks. The end of the study is defined as the last patient's last visit.

In case the study is ended prematurely, the investigator will notify the accredited METC within 15 days, including the reasons for the premature termination. Within one year after the end of the study, the investigator/sponsor will submit a final study report with the results of the study, including any publications/abstracts of the study, to he accredited METC.

6.10 Public disclosure and publication policy

The results of scientific research involving human subjects must be disclosed unreservedly and there are no objections on this regard.

7. REFERENCES

1. Swartz MS, Perkins DO, Stroup TS, et al. (2007). Effects of antipsychotic medications on psychosocial functioning in patients with chronic schizophrenia: findings from the NIMH CATIE study. Am J Psychiatry; 164 (3): 428-436

2. Staring ABP & van der Gaag M. Cognitieve gedragstherapie voor demoralisatie bij schizofrenie (article in Dutch). Gedragstherapie, 2010; 43: 205-224.

3. Grant PM, Huh GA, Perivoliotis D, et al. Randomized trial to evaluate the efficacy of cognitive therapy for low-functioning patients with schizophrenia. Arch Gen Psychiatry. 2012;69(2):121-127.

4. Verma S, Subramaniam M, Abdin E, et al. (2012). Symptomatic and functional remission in patients with first-episode psychosis. Acta Psychiatr Scand.;126(4):282-9.

5.Multidisciplinaire Richtlijn Schizofrenie, 2012 http://www.kwaliteitskoepel.nl/assets/structured-files/2012/ Richtlijn_Schizofrenie_20121.pdf

6. Staring AB, Ter Huurne MA, van der Gaag M. Cognitive behavioral therapy for negative symptoms (CBT-n) in psychotic disorders: A pilot study. J Behav Ther Exp Psychiatry. 2013;44(3):300-306.

7. Grant PM, Beck AT. (2010). Asocial beliefs as predictors of asocial behavior in schizophrenia. Psychiatry Res.;177(1-2):65-70.

8. Kay SR, Fiszbein A, Opler LA. The positive and negative symptom scale (PANSS). Schizophr Bull 1987; 13: 261– 76.

9. Myin-Germeys I, Oorschot M, Collip D, et al. (2009). Experience sampling research in psychopathology: opening the black box of daily life. Psychol Med.; 39(9):1533-47

10. Kirkpatrick B, Strauss GP, Nguyen L, et al. The brief negative symptom scale: psychometric properties. Schizophr Bull, 2011; 37(2):300-305.

11. Strauss GP, Keller WR, Buchanan RW, et al.. Next-generation negative symptom assessment for clinical trials: Validation of the Brief Negative Symptom Scale. Schizophr Res, 2012 ;142(1-3): 88-92.

12. http://www.euroqol.org

13. American Psychiatric Association, 1994. DSM-IV: Diagnostic and Statistical Manual of Mental Disorders, 4th ed. The Association, Washington, DC.

14. L. Hakkaart-van Roijen. Handleiding Short Form- Health and Labour Questionnaire (SF-HLQ). Rotterdam: iMTA, Erasmus Universiteit, 2010).

15. Addington D, Addington J, Maticka-Tyndale E. Assessing depression in schizophrenia: the Calgary Depression Scale.Br J Psychiatry Suppl. 1993 Dec;(22):39-44.

16. Putman P, Hermans E, van Honk J. Emotional stroop performance for masked angry faces: it's BAS, not BIS. Emotion 2004;4(3):305–11.

17. Lasalvia A, Bonetto C, Malchiodi F, et al. (2005) Listening to patients' needs to improve their subjective quality of life. Psychol Med 35:1655– 1665.

18. Hakkaart-van Roijen L. Tan SS, Bouwmans CAM. Handleiding voor kostenonderzoek: methoden en standaard kostprijzen voor economische evaluaties in de gezondheidszorg. Geactualiseerde versie 2010. Rotterdam: iMTA

19. Hakkaart-van Roijen L. Trimbos/iMTA Questionnaire Costs associated with Psychiatric illness (TiC-P). Rotterdam: iMTA, 2002.

20. Lamers LM, Stalmeier PFM, McDonnel J, et al. Kwaliteit van leven meten in economische evaluaties: het Nederlandse
EQ-5D tarief. Nederlands Tijdschrift Geneeskunde 2005;149:1574-8.

21. Mauskopf JA, Sullivian SD, Annemans L, et al. Principles of Good Practice for Budget Impact Analysis: Report of the ISPOR Task Force on Good Research Practices—Budget Impact Analysis. Value in Health 2007;10(5):336-347.

22. Briggs A, Claxton K, Sculpher M. Decision modelling for health economic evalutation. Oxford: Oxford University Press, 2006.

23. de Haan L, Klaassen R, van Beveren N, Wunderink L, Rutten BP, van Os J. Psychotic disorders: the need for staging. Tijdschr Psychiatr. 2012;54(11):927-33. Review. Dutch.

24. Hoch, J. (2009). Net benefit regression. In M. Kattan (Ed.), Encyclopedia of medical decision making. (pp. 806-812). Thousand Oaks, CA: SAGE Publications, Inc.
